# A NARRATIVE STUDY TO CONNECT THE GERONTOLOGY: THE ELDERLY CHARACTERS IN NURSING HOMES FIRE NEWS IN TAIWAN

**DOI:** 10.1093/geroni/igz038.713

**Published:** 2019-11-08

**Authors:** Sheng-Chin HSU

**Affiliations:** Ming Chuan University, Taipei, Taiwan

## Abstract

There are more than 30 civilian deaths in 10 fire accidents in nursing homes since 2008 in Taiwan. Due to the collective space of nursing homes, the damages of fires in a facility are much worse than in a personal house. Since the type of family changed after The Chinese Civil War, the elderly members, retired from the labor market, like to choose to live in nursing homes, or hospitals. According to the Ministry of Interior, R.O.C., Taiwan turned into an Aged society in 2018. In those fire news, the duties of families are seldom discussed, but the duties of care center are emphasized. The narrative of a fire accident in nursing homes is composed of many elements. These elements have been connected with old people and various arrangements. It is important to understand how the duties shifting from families to nursing homes, and what characters and relationship disappear in a fire accident. This study adopts the narrative theory to analyze the characters in fire report about Nursing Homes. Three findings are discovered: first, the duties of taking care of elders in a nursing home fire are connecting to Care Facilities but not families nor government. Second, the role of taking care of older people is played by Care-Staff more than family members in a fire narrative. Finally, the duty of fire and the duty of taking care of elders are interwoven into a complicated narrative: the role of Long-Term Care Center emerges but family and government disappear.

